# Supplementary material for: Interrogating the Metabolomic Profile of Amyotrophic Lateral Sclerosis in the Post-Mortem Human Brain by Infrared Matrix-Assisted Laser Desorption Electrospray Ionization (IR-MALDESI) Mass Spectrometry Imaging (MSI)
Source: Metabolites. 2022 Nov 10;12(11):1096. doi: 10.3390/metabo12111096 (PMC9696666; doi:10.3390/metabo12111096)
Supplement: Supplementary file 1 [file metabolites-12-01096-s001.zip › metabolites-1923695-supplementary.pdf]

Interrogating the Metabolomic Profile of Amyotrophic Lateral Sclerosis in the Post-Mortem Human Brain by Infrared Matrix-Assisted Laser Desorption Electrospray Ionization (IR-MALDESI) Mass Spectrometry Imaging (MSI)

Alexandria L. Sohn [1], Lingyan Ping [2,3,4], Jonathan D. Glass [3], Nicholas T. Seyfried [2,3,4], Emily C. Hector [5] and David C. Muddiman [1,6,*]

[1] FTMS Laboratory for Human Health Research, Department of Chemistry, North Carolina State University, Raleigh, NC 27695, USA
[2] Goizueta Alzheimer's Disease Research Center, Emory University School of Medicine, Atlanta, GA 30322, USA
[3] Department of Neurology, Emory University School of Medicine, Atlanta, GA 30322, USA
[4] Department of Biochemistry, Emory University School of Medicine, Atlanta, GA 30322, USA
[5] Department of Statistics, North Carolina State University, Raleigh, NC 27695, USA
[6] Molecular Education, Technology and Research Innovation Center (METRIC), North Carolina State University, Raleigh, NC 27695, USA
* Correspondence: dcmuddim@ncsu.edu; Tel.: +1-919-513-0084

Supporting Information

Submitted to: *Metabolites*

Accepted: 07 November 2022

Manuscript: 6 Pages / 3 Supplemental Figures / 3 Supplemental Tables

Keywords: IR-MALDESI MSI; mass spectrometry imaging; Amyotrophic lateral sclerosis (ALS); neurodegenerative disease; multiomic

Table of Contents

Figure S1. Arginine biosynthesis pathway annotated with ion images and protein detection between metabolomic conversions.

Figure S2. Examples of metabolite conversions detected by IR-MALDESI with associated proteins reported in a previous LC-MS/MS study by the Seyfried group.

Figure S3. Experimental isotopic distributions of detected metabolites reported in **Figures 8** and **S2**.

Table S1. Patient case information for frontal cortex samples analyzed via IR-MALDESI.

Table S2. Cumulative list of putative metabolite identifications from brain sections imaged by IR-MALDESI MSI.

Table S3. Metabolites indicated as statistically significant or a significant fold-change from volcano plots in **Figure 3.**

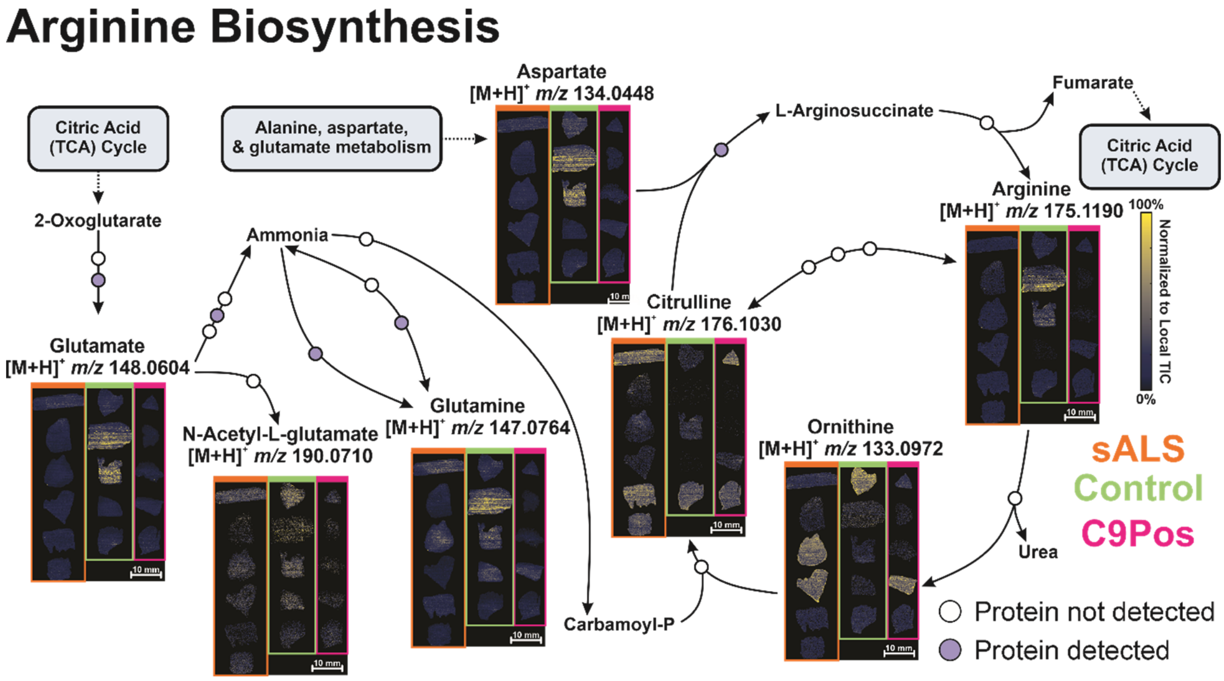

Figure S1. Arginine biosynthesis pathway annotated with associated metabolite ion images by IR-MALDESI. Glutamate was found to be dysregulated between disease classifications and could cause disruption in the synthesis of arginine. Circles along conversion indicate the quantity of proteins involved in metabolomic conversion as reported by KEGG database, and color is indicative of detection or lack thereof. Each disease classification is indicated by the color of the box surrounding the samples (bottom right).

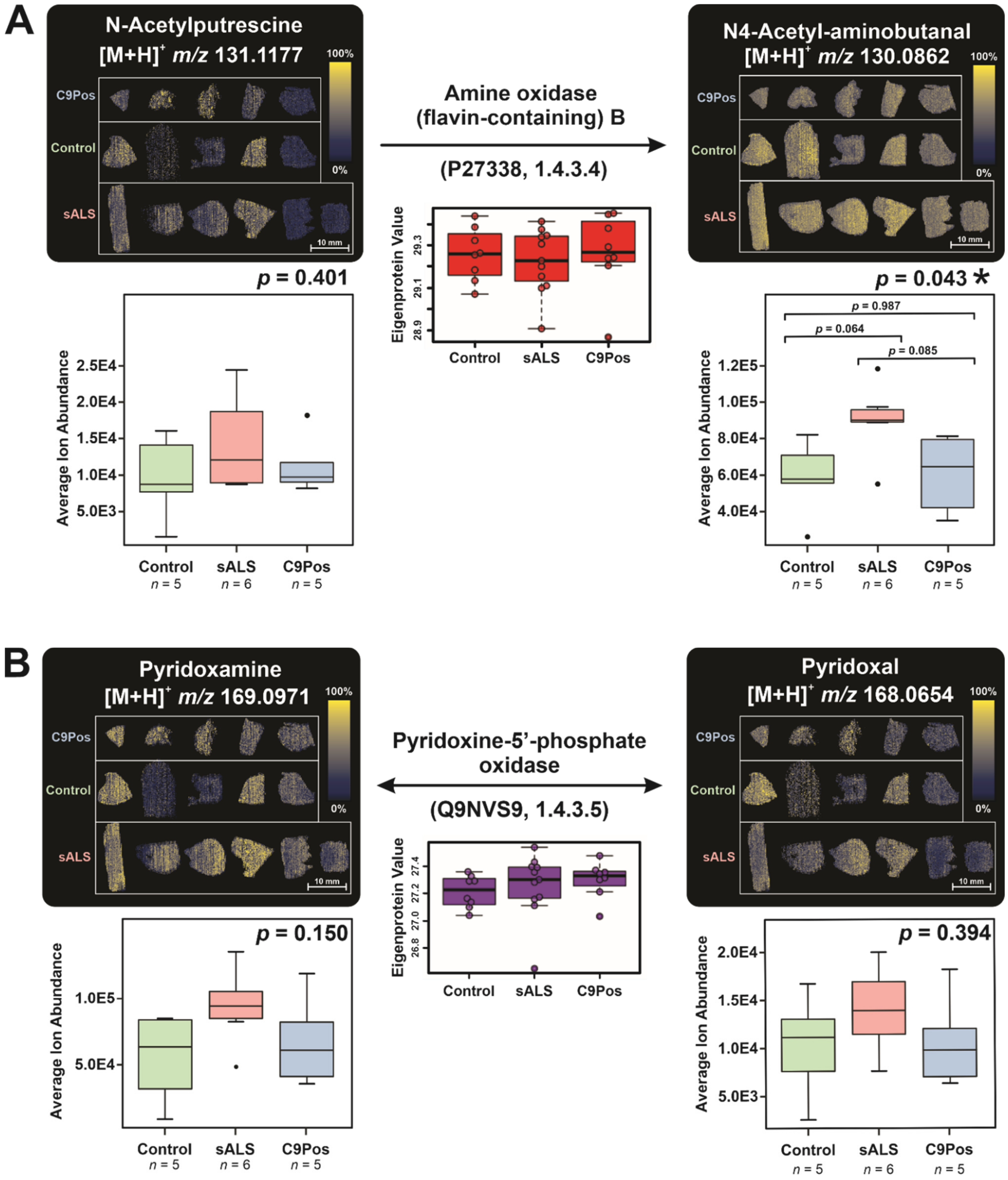
A
N-Acetylputrescine
[M+H]+ m/z 131.1177
Amine oxidase
(flavin-containing) B
(P27338, 1.4.3.4)
N4-Acetyl-aminobutanal
[M+H]+ m/z 130.0862
C9Pos
Control
sALS
100%
0%
10 mm
p = 0.401
p = 0.043 *
p = 0.987
p = 0.064
p = 0.085
Eigenprotein Value
Average Ion Abundance
Control
sALS
C9Pos
n = 5
n = 6
n = 5
B
Pyridoxamine
[M+H]+ m/z 169.0971
Pyridoxine-5'-phosphate
oxidase
(Q9NVS9, 1.4.3.5)
Pyridoxal
[M+H]+ m/z 168.0654
p = 0.150
p = 0.394

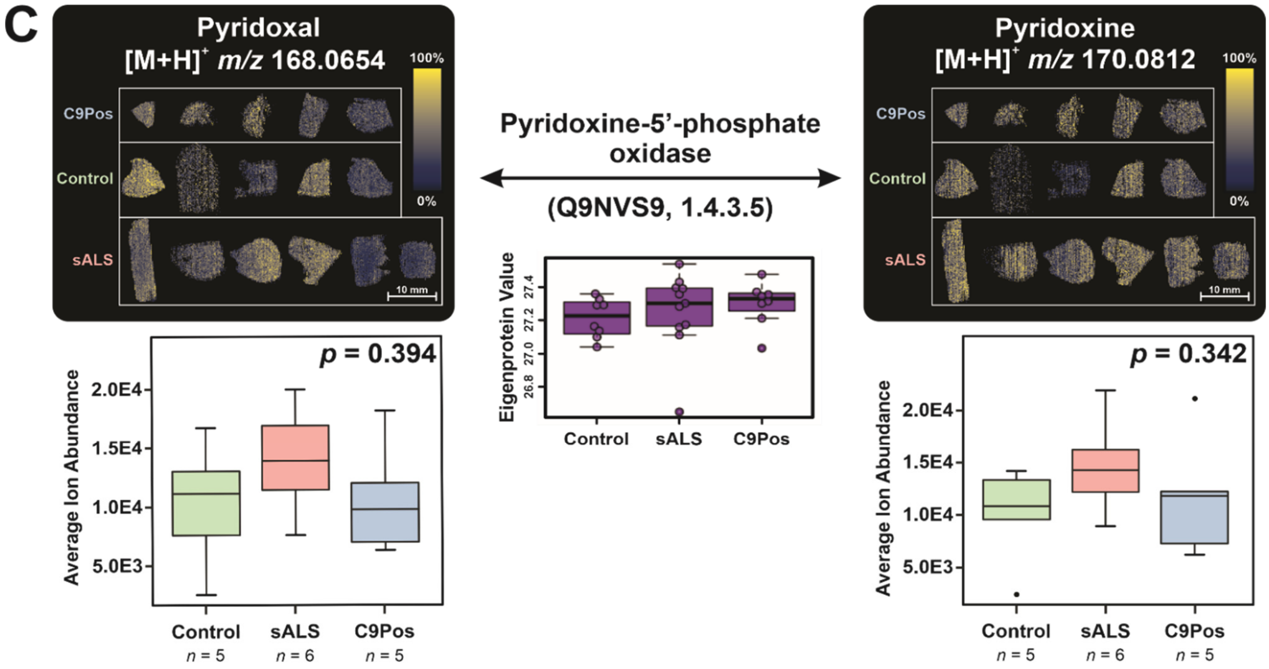

Figure S2. Three additional examples of metabolite conversions by the respective intermediate proteins. Ion images represent metabolomic detection by IR-MALDESI from post-mortem brain samples separated by disease classification, C9Pos, control, and sALS (respectively blue, green, and pink). Ion images are accompanied with boxplots of average ion abundance for each sample and *p*-values from one-way ANOVA testing. Significant results were found with *m/z* 130.0862 (**A**, right), so further testing by Tukey's HSD tests for pairwise comparisons were performed. Brackets are above each combination of cohort to show *p*-value associated from Tukey's tests in addition to the results from ANOVA. The protein involved in conversion is shown with its Uniprot accession ID and EC number.

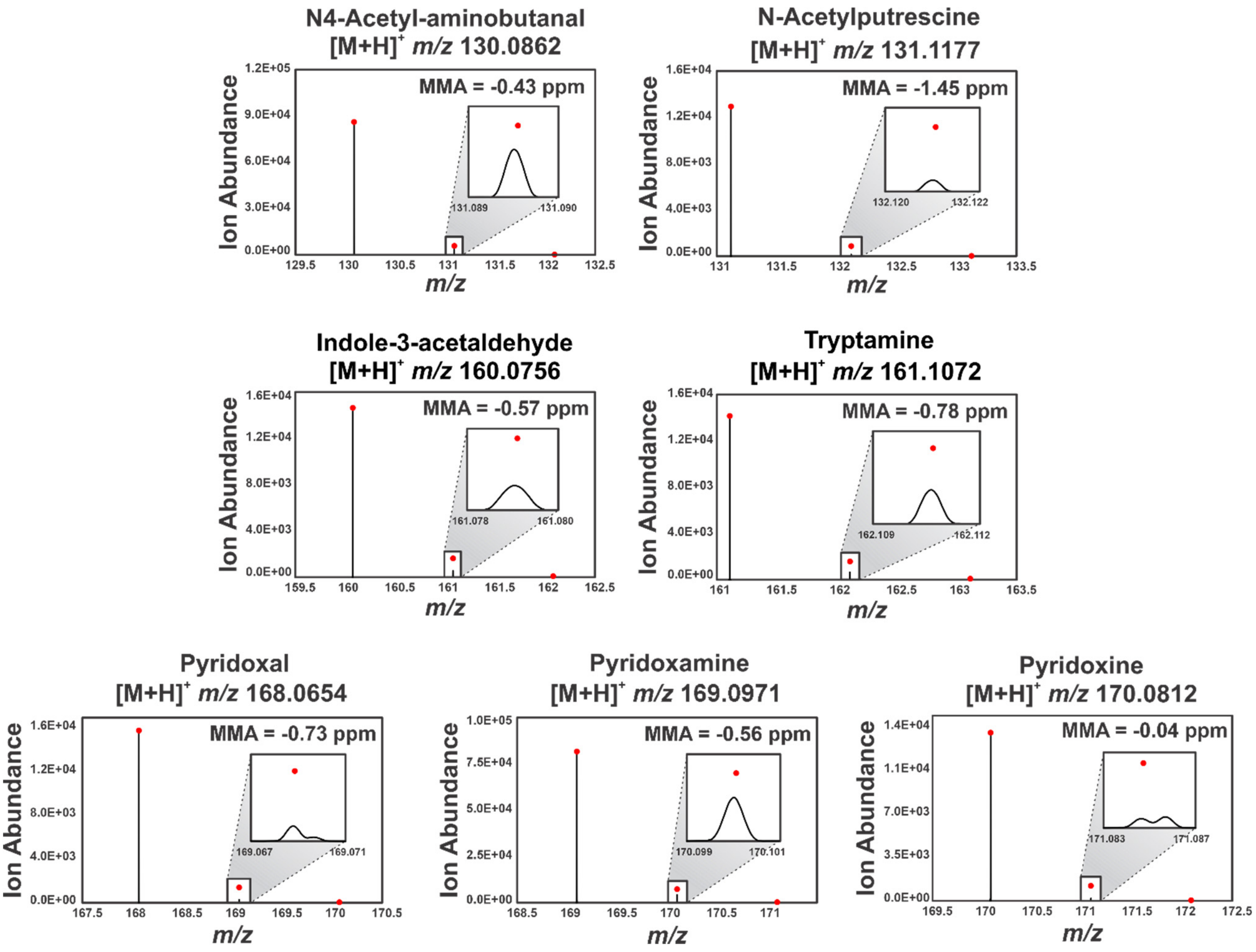

Figure S3. Experimental isotopic distributions for metabolite conversions from **Figures 8 and S2**. Experimental *m/z* values are reported with the respective mass measurement accuracy (MMA) in parts-per-million.
